# Stability Study and a 14-Day Oral Dose Toxicity in Rats of Plantain Leaf Extract (*Plantago lanceolata* L.) Syrup

**DOI:** 10.3390/scipharm85010015

**Published:** 2017-03-22

**Authors:** Kenza Mansoor, Fadi Qadan, Mathias Schmidt, Eyad Mallah, Wael Abudayyih, Khalid Matalka

**Affiliations:** 1 Department of Pharmaceutical Medicinal Chemistry and Pharmacognosy, Faculty of Pharmacy & Medical Sciences, University of Petra, 11196 Amman, Jordan; emallah@uop.edu.jo (E.M.); wabudayyih@uop.edu.jo (W.A.); 2 Herbresearch Germany, 86874 Mattsies, Germany; fqadan@googlemail.com (F.Q.); schmidt@herbresearch.de (M.S.); 3 Department of Medical Laboratories, Faculty of Health Sciences, American University of Madaba, 11821 Madaba, Jordan; k.matalka@aum.edu.jo

**Keywords:** oral toxicity, plantain (*Plantago lanceolata* L.), stability testing, cough, common cold

## Abstract

Plants have been used since antiquity to treat and prevent diseases. Plantain (*Plantago lanceolata* L.) is traditionally used for the treatment of the common cold and associated symptoms such as cough. This study was designed to evaluate the oral toxicity of plantain leaf extract-containing syrup. In preparation of the toxicological examination and to ensure the quality of the herbal preparation, analytical methods were developed and validated, and stability testing was performed. Physicochemical and microbial quality, thin layer chromatography patterns and high performance liquid chromatography fingerprints complied with the specifications during the entire period of stability testing. The marker substance, acteoside, remained within the stability-defining limits of 90%–110% for quantitative determinations. No hint of toxicity emerged from 14-day repeat dose toxicity testing in rats. The animals were given doses of 3, 6, or 12 mL of syrup per kg body weight by gavage twice daily. All animals showed normal appearance and behavior. Body and organ weights at the end of the study were similar to those in the control group. Overall, *P. lanceolata* syrup was found to be stable and non-toxic under the test conditions.

## 1. Introduction

Herbal medicinal products are enjoying increasing popularity. Unfortunately, many plant-derived products used for therapeutic purposes do not meet the current standards for herbal drugs and are therefore not fully evaluated for their quality, safety and efficacy [1]. The European Medicines Agency (EMA) is setting standards for the establishment of an appropriate quality of natural remedies. The regulatory concern is that plants are not necessarily safe only because they are natural, as many potentially toxic constit4uents may be present in plant material, naturally or as contaminants. The development of herbal medicinal products therefore requires tests for quality and safety [2].

Species of the botanical gender *Plantago* (Plantaginaceae) with its approximately 275 taxa [3] belong to the most widely used medicinal plants on a worldwide scale [4]. Ethnopharmacological studies have confirmed the traditional use of *Plantago* species for the treatment of skin infections and digestive, respiratory, reproduction and circulatory disorders, the application against tumors, for pain relief and fever reduction [5]. Through phytochemical analyses of *Plantago* species, the presence of a range of chemical constituents such as alkaloids, caffeic acid derivatives, coumarins, fats and oils, phenolics (flavonoids, tannins), iridoids, mucilage, polysaccharides, sterols and volatile compounds was demonstrated [3,4,6].

*Plantago lanceolata* (plantain), as one of the major species used as a medicinal plant, is commonly used in the treatment of common colds [7] and infections of the respiratory system [8,9], to soothe and suppress cough [6,9,10,11], or as an antiviral [3], antimicrobial [3,12], anti-inflammatory [3,6,13,14] or antioxidant [3,13,15,16] agent. Topically applied, plantain is traditionally used for wound healing [6,15] and draining abscesses [15,17]. The major phytochemical constituents of potential clinical importance are phenolics, predominantly comprised of flavonoids and hydroxycinnamic acid derivatives [13,15].

The EMA confirmed the traditional use against the common cold and cough in a Community Herbal Monograph [18]. Although the 30-year rule of exposure for traditional herbal medicinal preparation serves as a surrogate for toxicity studies, there is actually only limited experience from toxicology, which may impede the development of evidence-based medicinal products through clinical studies in old and new indications. 

The background for this work was the development of a plantain syrup with adequate quality and safety. For the study presented herein, a syrup containing plantain leaf extract was developed together with appropriate validated methods for quality assurance. The newly developed analytical methods allowed the performance of stability and toxicity testing carried out in line with the relevant guidelines of the International Council for Harmonisation of Technical Requirements for Pharmaceuticals for Human Use (ICH), of the United States Environmental Protection Agency/Office for Pollution Prevention and Toxics (US EPA/OPPTS), and of the Organisation for Economic Co-operation and Development (OECD).

## 2. Materials and Methods

### 2.1. Solvents

Solvents for chromatography were purchased from Acros Organics, Morris Plains, NJ, USA.

### 2.2. Dry Plantain Leaves

Dry plantain leaf material was purchased from Martin Bauer (Vestenbergsgreuth, Germany). 

### 2.3. Plant Extract

Standardized *Plantago lanceolata* dry leaf extract (Extract No. 0174300, DER 3-5:1; 70% native, extraction solvent 20% ethanol *v*/*v*) was donated by Finzelberg GmbH, Sinzig, Germany. A sample was deposited at the Department of Pharmaceutical Medicinal Chemistry and Pharmacognosy, University of Petra, Amman, Jordan (voucher number: EX032017). Authentication and identification of the extract was carried out by thin layer chromatography [19,20], following the method defined for Plantaginis lanceolatae herba of the European Pharmacopoeia. The mobile phase was acetic acid: anhydrous formic acid:water:ethyl acetate 11:11:27:100. Aucubin (Sigma-Aldrich, Munich, Germany) was used as a reference substance. 

### 2.4. Syrup Preparation

*Plantago* syrup was formulated with the composition detailed in Table 1. The dry extract preparation was completely soluble in the syrup formulation. The quantity of preservative was selected based on microbiological challenge tests during formulation development (not reported herein). The syrup was stored at ambient temperature in amber glass bottles. A placebo preparation was prepared as a control for the toxicological study, using the same composition with the exception of plantain dry extract. Two production batches of the plantain syrup with 1000 L each and a pilot batch of 100 L were prepared.

### 2.5. Physicochemical Quality Control of the Liquid Preparations

Quality control of the syrup involved organoleptic characteristics (appearance, odor, and taste in comparison with previously prepared samples) and physicochemical properties: pH was measured according to Ph. Eur. 2.2.3 using a Mettler Toledo pH meter (Mettler Toledo, Columbus, OH, USA) [20]. Relative density was measured according to Ph. Eur. 2.2.5 [20], and filling volume was evaluated volumetrically.

### 2.6. High Performance Thin Layer Chromatography (HPTLC) Method for Quality Control

The HPTLC method for the identification of plantain dry extract in the formulation was based on Silicagel 60 F254 HPTLC plates as the stationary phase, and ethyl acetate:methanol:water 7:2:1 as the mobile phase. The test solution was prepared by dissolving 5 mL of cough syrup in 5 mL methanol, followed by sonication for 15 min and centrifugation at 16,000 rpm (28,672× *g*) for 5 min. The placebo test solution was prepared by dissolving 20 mg of placebo syrup (excipient mixture of the finished product) in the same way as the test solution. Herbal drug material as a reference was extracted with 20% ethanol (50:1, *w*/*v*) for 15 min using a sonicator for 15 min. The extract was centrifuged at 16,000 rpm (28,672× *g*) for 5 min and the supernatant was tested in the HPTLC as such. Plantain dry extract reference solution was prepared by dissolving 70 mg of extract in 5 mL methanol. Aucubin (0.2 mg/mL; Sigma-Aldrich) and naphthol yellow (1 mg/mL; Sigma-Aldrich) were used as standard reference compounds dissolved in methanol.

Dimethylaminobenzaldehyde reagent (0.2 g in a mixture of 4.5 mL water and 5.5 mL hydrochloric acid 36%) was used as a spraying reagent after development of the plate and drying. Evaluation of the HPTLC plates took place at daylight and after spraying.

### 2.7. High-Performance Liquid Chromatography Assay: Determination of Extract Content in the Syrup

The analytical high-performance liquid chromatography (HPLC) system consisted of a Merck KGaA (Darmstadt, Germany) high-performance liquid chromatograph coupled with an UV-detector (UV-Detector L-4250). Analytical data were evaluated using a Merck KGaA data processing system (D-7000). Separation was achieved on a Merck Superspher RP 18, 250 × 4 mm column at 40 °C. The mobile phase consisted of methanol (solvent A) and methanol/0.02 M H_3_PO_4_ (5/95 *v*/*v*) (solvent B), using the gradient program listed in Table 2. 

The flow rate was 1 mL/min, and the injection volume was 10 µL. The wavelength was 203 nm (0–12 min) and 330 nm (12–27 min). The identification of each compound was based on a combination of retention time and spectral matching. The method was validated for system suitability, linearity, accuracy (recovery) and precision.

The determination of the quantity of extract in the syrup was based on the quantification of the constituent acteoside in the batch-specific extract. The *Plantago* dry extract standard solution was prepared by dissolving 215 mg of extract in 100 mL of 80% methanol. Triplicate samples were analyzed and the average peak area of acteoside was determined using caffeic acid as an external standard (20 mg dissolved in 100 mL methanol 80% *v*/*v*; triplicate measurements).

An amount of 25 mL of the test solution, described in the section on the HPTLC method, was diluted to 80 mL with methanol 80% *v*/*v*. This solution was sonicated for 5 min at 20 °C, and filled up to 200 mL. Triplicate samples of this solution were analyzed and the average peak area of acteoside was determined. Extract content in the syrup was then calculated referring to the previously determined acteoside content in the extract. For reasons of simplicity and a better documentation of stability, the results section states the findings for acteoside instead of the extract quantity.

### 2.8. Quantification of Preservative Potassium Sorbate in the Syrup by High-Performance Liquid Chromatography

The assay on potassium sorbate was achieved at ambient temperature on a Nucleosil column 100-5 C18; 100 × 4.6 mm. The isocratic mobile phase consisted of acetonitrile/acetate buffer. The flow rate was 1 mL/min, and the injected volume was 20 µL. The monitoring wavelength was 240 nm for 20 min. Batch-specific potassium sorbate was used as an external working standard: 30 mg potassium sorbate (exactly weighed) was dissolved in 100 mL of water and sonicated in an ultrasonic bath for 5 min. The test sample was prepared by diluting 5 mL of the syrup in 50 mL water. Triple tests were carried out for the standard and for the test solution.

### 2.9. Determination of Microbiological Purity

The specifications for microbiological purity corresponded to the definitions of Ph. Eur. 5.1.8., Category B. The procedure involved serial dilution of samples. The spread plate technique was used to enumerate the microbial contaminant from the collected syrup samples. One milliliter from each sample was withdrawn aseptically and transferred into 9 mL normal saline for serial dilution. Diluted samples were thoroughly mixed for the proper dissolution of the drug. 

About 0.1 mL of each diluted sample was spread aseptically onto nutrient agar, mannitol salt agar (MSA), MacConkey agar and membrane fecal coliform (mFC) agar media plates for the enumeration of total viable bacteria, *Staphylococcus aureus, Escherichia coli*, and total coliforms, respectively. Inoculated plates were incubated for 24 h at 37 °C. The number of colony forming units (CFU) was counted manually per milliliter of the syrup sample.

Further microorganisms were determined using different methods: 1 mL of the test sample was added to 9 mL of alkaline peptone water and incubated for 4–6 h at 37 °C. The 1–3 loop full sample was streaked on thiosulfate-citrate-bile salts-sucrose agar (TCBS agar) plates and incubated at 37 °C for 18–24 h. Sucrose fermenting colonies were further identified using standard biochemical tests. About a 1 mL sample was added to 9 mL of Selenite Cysteine Broth and incubated for 4–6 h at 37 °C. The enriched sample was streaked on *Salmonella Shigella* Agar (SS agar) plates and incubated at 37 °C for 18–24 h.

### 2.10. Stability Study

Stability studies were carried out with the plantain syrup according to ICH guidelines at 25 °C/60% relative humidity (R.H.) for two years and at 40 °C/45% R.H. Storage was made in electronically monitored stability chambers (Binder, Tuttlingen, Germany). Stability testing was started immediately after release of the batches.

### 2.11. Repeated Dose Oral Toxicity Study

Female albino rats were accommodated in a 12 h light–dark cycle at a temperature of 20 ± 2 °C and acclimated to laboratory conditions for two weeks without showing any abnormality or pathological changes prior to experiments. Twenty rats weighing between 150 and 210 g were randomly divided into four groups; each containing five rats. The first group was the control which received the placebo formulation (see Table 1), whereas the other three groups were orally administered 3, 6 and 12 mL/kg *Plantago* syrup by gavage twice daily for 14 days.

Meanwhile, free access to a commercial rodent diet (Research Diets INC, New Brunswick, NJ, USA) and water was allowed, except when fasting was needed in the course of the study (12 h before dosing). Experiments were performed according to the Guide for the Care and Use of Laboratory Animals (published by the US National Institutes of Health NIH Publication no. 85–23, revised 1996). Furthermore, the study protocol was revised and approved by the Ethical Committee of the Faculty of Pharmacy and Medical Sciences, University of Petra, Amman, Jordan and was given number Eth.UOP 2015/10. 

### 2.12. Clinical Monitoring, Gross Pathological Examination and Organ Weights

All study groups were thoroughly monitored during and after dose administration, with a focus on the observation and reporting of adverse effects, behavioral changes or deaths that might occur during the post-dose period. A daily investigation and examination was carried out for a period of fourteen days (study period). Body weight was recorded at the beginning and at the end of the study. Animals were sacrificed at the end of study period. The major organs (spleen, liver, lungs and kidneys) were isolated, dissected and visually evaluated for any pathological changes in comparison with the control group. Kidneys and livers were accurately weighed and recorded.

### 2.13. Statistical Analysis

All data were analyzed by one-way analysis of variance (Minitab 14, Minitab Inc, Coventry, United Kingdom). Differences were considered significant at *p < 0.05*, and data were presented as means ± SD (Standard Deviation).

## 3. Results

An HPLC method was developed and validated according to the definitions of ICH guideline Q2 (Validation of analytical procedures) [21] for the quantification of plantain extract in plantain syrup, as a precondition for stability studies, which by themselves served as a foundation for the performance of a repeated-dose toxicity study. The method was found suitable, accurate and precise. 

No changes to the formulation were found in long-term stability testing at 25 °C/60% R.H. for the duration of 24 months (data not shown), and in accelerated stability testing with six months storage at 40 °C/75% R.H. as recommended by ICH guideline Q1A (R2) (Stability testing of new drug substances and products) [22]. Table 3 shows the specifications and results of accelerated stability testing after three and six months as the worst-case model in stability testing. The results confirm at least 24 months of stability, with a labelling stating that no special storage conditions are required for the herbal preparation.

### 3.1. Identity

**Figure 1:**
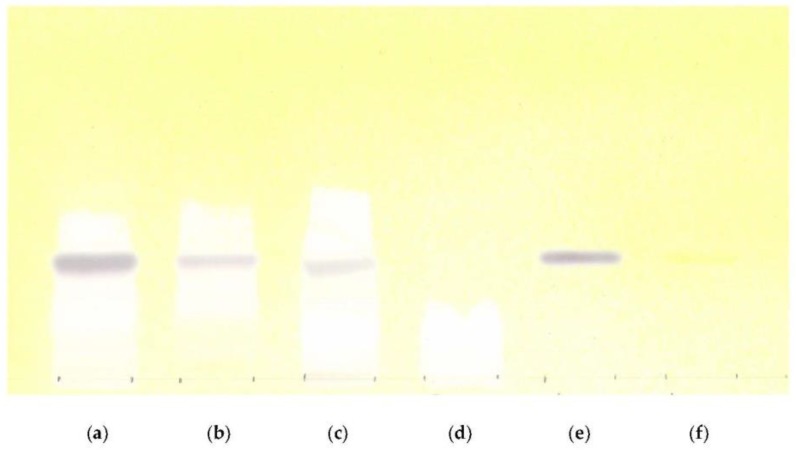
TLC results: (**a**) *Plantago lanceolata* leaf; (**b**) *Plantago lanceolata* dry extract; (**c**) *Plantago* syrup; (**d**) Placebo; (**e**) Aucubin standard; (**f**) TLC marker naphthol yellow.

Identity was tested using thin layer chromatograhpy (TLC) and HPLC at the beginning and during the stability of batches. TLC chromatograms obtained with the test and reference solutions showed a brown-grey zone corresponding to aucubin at the same height as the zone of reference aucubin in the chromatogram. The zone of aucubin is also at the same height as the yellow zone of the TLC marker naphthol yellow (nearly invisible). In the chromatogram of the placebo formulation, no zones were detected (Figure 1). During the long-term and accelerated stability study of different batches, no changes were observed in the chromatograms of the tested samples.

### 3.2. Assay on acteoside as a stability marker of Plantago extract

**Figure 2:**
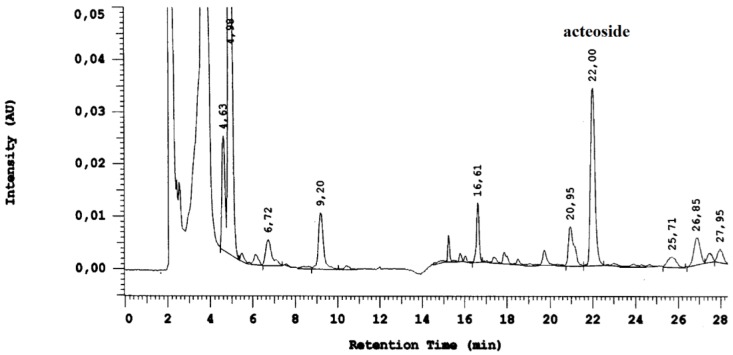
High-performance liquid chromatography chromatogram of the finished product “*Plantago* syrup”.

According to the European Pharmacopeia monograph “plantaginis lanceolatae folium”, the content of acteoside should not be less than 1.5% [20]. This requirement justifies the selection of acteoside as a quality marker for plantain syrup. Acteoside was identified and quantified in the samples by HPLC. The average retention time of acteoside was 22.00 min (Figure 2).

The formulation was found stable in long-term and accelerated stability testing (for the latter see Table 2) in accordance with ICH guidelines [22].

### 3.3. Microbiological Purity

The microbiological testing was undertaken in accordance with the specifications in the European Pharmacopeia. None of the tested samples were microbiologically contaminated (Table 3). Through additional testing beyond the specifications of Ph. Eur. 5.1.8. Category B for herbal medicinal products [20], all samples were found free from *Vibrio* *cholerae*, *Staphylococcus aureus* and *Shigella* spp.

In addition to testing the microbiological properties, the concentration of potassium sorbate (preservative) was tested throughout the stability study. It was likewise found stable and yielding sufficient protection against microbiological contamination (Table 3).

### 3.4. Repeated Dose 14-Day Oral Toxicity Study with Plantago Syrup

**Figure 3:**
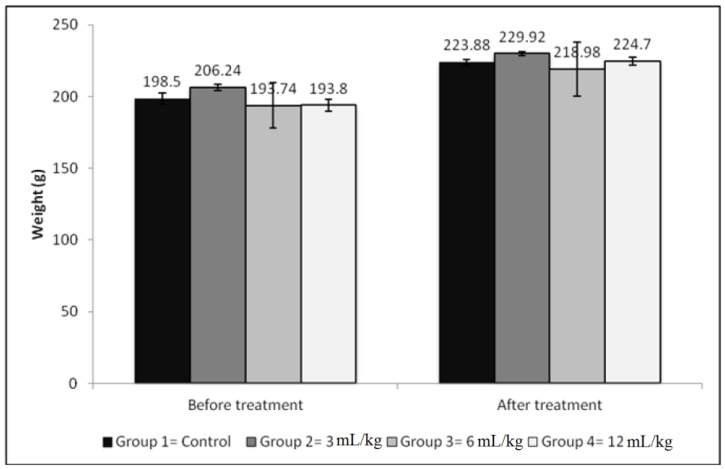
Body weight changes before and after 14-days treatment with *Plantago* syrup. The treated and controlled groups showed normal weight gain. There were no significant differences in average weights between the treated groups and control groups (*p* > 0.05). Values represent the average weight of five rats ± SD.

All animals stayed alive and appeared normal without any clinical abnormality at the end of the observation interval. All clinical findings of the treated animals were not different from those of the control group. The rats behaved normally with no signs of illness and discomfort. There was no significant difference (*p* > 0.05) in average body weights among the four test groups. Food and water consumption was normal. Moreover, a similar weight gain was observed for control and treated groups (Figure 3). None of the major organs, namely, spleen, liver, lungs and kidneys, showed any gross anatomical abnormalities. The differences in the kidneys and liver weights before and after the repeated dose toxicity study were not significant in comparison with control groups (*p* > 0.05) (Table 4).

## 4. Discussion

*Plantago lanceolata* dry extract is a complex mixture of phytochemical substances [23]. At present, the pharmacological effect of the drug cannot be definitively ascribed to one single constituent or a group of compounds. One of the main constituents in *Plantago lanceolata* is the caffeic acid ester acteoside (also known as verbascoside). Further characteristic constituents are the iridoid monoterpenes aucubin and catalpol, isoacteoside and chlorogenic acid. As long as there is no definitive association between clinically observed efficacy and an identified constituent of fraction, a standardization to a defined content of this substance or fraction is not possible. The definition of a minimum content of dihydroxycinammic acid derivatives in plantain herb (calculated as acteoside) according to the European Pharmacopoeia monograph is merely a marker for the assurance of sufficiently well-defined quality. It was therefore selected as a marker compound for plantain extract and plantain syrup as well. The content of acteoside in the syrup was within the range of 90% and 110% at all points during the long-term and the accelerated stability study.

The quantity of the preservative potassium sorbate had been defined during formulation development based on microbiological challenge tests. The content of the preservative is an important parameter in stability testing. Even under the relatively harsh conditions of accelerated stability testing at 40 °C/75% R.H., the quantity after six months was still 97.7% of the starting value — well within the specified range of 80% to 120%. The accelerated stability testing confirmed the findings of 24 months of long-term stability testing and the labelling of no specific storage conditions required for the product.

The stability results assured the quality of *Plantago* syrup during the repeated dose toxicity testing. This 14-day oral testing by gavage applied daily doses of up to 24 mL of syrup. This dose translates into a theoretical exposure to 1200 mL of syrup in a human of 50 kg body weight. At a concentration of 2.5% of extract preparation, the 1200 mL would contain 30 g of plantain dry extract formulation. As this extract is an extract preparation with 70% native extract and 30% excipients, the exposure to native plantain dry extract would correspond to 21 g of native extract. The EMA’s Community Herbal Monograph on plantain leaves formulates a traditional dose recommendation of 700 mg of a relatively similar dry extract as the daily dose [18]. Accordingly, the high dose applied to the animals was 30 times higher than the recommended human dose. Usually, the dosing in animals would have to be oriented at the occurrence of target organ toxicity (in this case, the pulmonary system), but in the case of plantain the upper limit in toxicity testing is already reached through the limitations of the technical applicability of syrup by gavage, and even then there was still no sign of toxicity in any organ. The dosing in the toxicity study may therefore be considered sufficiently justified.

The EMA’s Community Herbal Monograph on the traditional use of plantain as a herbal medicinal product does not state any known adverse effects or contraindications beyond the usual warning against the possibility of hypersensitivity [18]. In fact, there seems to be only the report of Ozkol et al., 2012 on two suspected cases of phototoxic reactions after oral consumption of *P. lanceolata* preparations followed by exposure to sunlight [17]. Such cases have never been previously reported in the literature and from clinical experience. Phototoxicity would not be covered by our study design, but appears rather unlikely in view of the worldwide experience with plantain exposure. In the present study, *Plantago lanceolata* extract showed no signs of toxicity in rats in oral administration as syrup within the specified dose. This finding is in line with literature data: Ruiz et al., 1996; Lim-Sylianco and Shier, 1985 and Angelov et al., 1980 have found that *Plantago* was not toxic [24,25,26].

## 5. Conclusions

The developed HPLC analytical method was sufficiently suitable, accurate and precise, and can thus be used for quality control purposes of *Plantago* containing formulations. Furthermore, the formulation of *P. lanceolata* syrup was found to be stable for the duration of two years under long-term conditions and six months under accelerated conditions. Even with 30-fold of the recommended human dose, there was no sign of toxicity after 14 days of oral administration. We therefore conclude that the administration of products containing *Plantago* is likely to be safe, especially as the use against the common cold would usually involve a duration of exposures corresponding to the duration of repeated dose toxicity testing presented in this study.

## Figures and Tables

**Table 1:** Composition of *Plantago lanceolata* syrup (100 mL).

Ingredient	Function	Quantity per 100 mL	Supplier
*Plantago lanceolata* dry extract	Active constituent	2.5 g	Finzelberg, Andernach, Germany
Maltitol	Sweetener	40 g	Roquette, Lesterm, France
Potassium sorbate	Preservative	300 mg	Chemicalpoint, Oberhaching, Germany
Citric acid	pH adjuster	44 mg	Merck, Darmstadt, Germany
Honey flavor	Flavoring agent	100 mg	Symrise, Holzminden, Germany
Purified water	Solvent	Add to 100 mL	

**Table 2:** Gradient program for the high-performance liquid chromatography (HPLC) method.

Time	Solvent A	Solvent B
0–10 min	0%	100%
10–24 min	34%	66%
24–30 min	90%	10%
30–50 min	0%	100%

**Table 3:** Specifications and results of accelerated stability testing of *Plantago* syrup.

	Specification	Start	3 Months	6 Months
		14.08	14.11	15.02
Appearance	Dark brown clear liquid	Conforming	Conforming	Conforming
Taste	*Plantago*-typical, honey taste	Conforming	Conforming	Conforming
pH	4–5.5	4.8	4.4	4.8
Microbial quality	TAMC ≤10^4^/mL	<160	n.d. ^1^	<10
[Ph. Eur. 5.1.8 Cat. B] [20]	TYMC ≤10^2^/mL	<10	n.d. ^1^	<10
	Enterobacteria ≤10^2^/mL	<1	n.d. ^1^	<1
	*E. coli* absent in 1 mL	Absent	n.d. ^1^	absent
	*Salmonella* absent in 25 mL	Absent	n.d. ^1^	absent
	*S. aureus* <1/g			
Identity by TLC	Complies with the chromatogram at start point	Conforming with reference TLC	No changes	No changes
Identity by HPLC	Complies with the chromatogram at start point	Conforming with reference TLC	No changes	No changes
Assay	Acteoside (μg/mL)	54.32	53.92	53.88
	(calculated as caffeic acid)	100%	99.3%	99.19%
	[90%–110%]			
	Potassium sorbate (mg)	300	298.2	293.2
	[80%–120%]	100%	99.4%	97.7%
Efficacy of antimicrobial preservation	Ph. Eur. 5.1.3 [20]	Test passed	Test passed	Test passed
**Conclusions on stability**			**Stable**	**Stable**

^1^ n.d. = not determined; TAMC: Total Aerobic Microbial Count; TYMC: Total Yeast and Mould Count; TLC: Thin Layer Chromatography

**Table 4:** Weight of both kidneys and liver in control and 14-day treated groups with *Plantago* syrup.

	Weight (g)			
Organ	Group 1 (Control)	Group 2 (3 mL/kg)	Group 3 (6 mL/kg)	Group 4 (12 mL/kg)
Kidneys	2.18 ± 0.19	2.5 ± 0.22	2.48 ± 0.24	2.36 ± 0.31
Liver	11.64 ± 1.6	14.14 ± 1.7	13.58 ± 1.5	11.46 ± 2.1

Values show the average weight of each group (*n* = 5) ± SD. There was no statistical difference between treated groups and the control group (*p > 0.05*).
